# A multi-centre, participant-blinded, randomized, 3-year study to compare the efficacy of Virtual Surgical Planning (VSP) to Freehand Surgery (FHS) on bony union and quality of life outcomes for mandibular reconstruction with fibular and scapular free flaps: study protocol for a randomized phase II/III trial

**DOI:** 10.1186/s12885-025-13505-5

**Published:** 2025-02-27

**Authors:** Khanh Linh Tran, Sena Turkdogan, Anat Bahat Dinur, Thomas D. Milner, Edward Wang, Anthony Nichols, Danielle MacNeil, Adrian Mendez, Jake Jervis-Bardy, John De Almeida, Christopher Yao, David Goldstein, Ralph Gilbert, Antoine Eskander, Kevin Higgins, Danny Enepekides, Michael Gupta, Han Zhang, Michael Au, Sally Nguyen, Sidney Fels, Antony Hodgson, Penelope Brasher, Craig Mitton, Farahna Sabiq, Charles Fisher, David Yang, Angela Wong, Cathie Garnis, Catherine Poh, J. Scott Durham, Eitan Prisman

**Affiliations:** 1 Division of Otolaryngology, Department of Surgery, Faculty of Medicine, University of British Columbia, Vancouver, BC Canada; 2 Department of Otolaryngology– Head and Neck Surgery, Western University, London, ON Canada; 3 Department of Otolaryngology– Head and Neck Surgery, University Health Network, University of Toronto, Toronto, ON Canada; 4 Department of Otolaryngology– Head and Neck Surgery, Sunnybrook Hospital, University of Toronto, Toronto, ON Canada; 5 Division of Otolaryngology– Head and Neck Surgery, McMaster University, Hamilton, ON Canada; 6 Department of Otolaryngology– Head and Neck Surgery, University of Ottawa, Ottawa, ON Canada; 7 Department of Electrical and Computer Engineering, Faculty of Applied Science, University of British Columbia, Vancouver, BC Canada; 8 Department of Mechancial Engineering, Faculty of Applied Science, University of British Columbia, Vancouver, BC Canada; 9 Centre for Clinical Epidemiology and Evaluation, Vancouver Coastal Health Research Institute, Vancouver, BC Canada; 10 School of Population and Public Health, Faculty of Medicine, University of British Columbia, Vancouver, BC Canada; 11 Department of Radiology, Faculty of Medicine, University of British Columbia, Vancouver, BC Canada; 12 Department of Orthopaedics, Faculty of Medicine, University of British Columbia, Vancouver, BC Canada; 13 Faculty of Dentistry, University of British Columbia, Vancouver, BC Canada

**Keywords:** Head and neck cancer, Oral cavity, Mandible Reconstruction, Virtual Surgical Planning, Quality of life, Randomized controlled trial

## Abstract

**Background:**

Advanced head and neck malignancies with underlying bony involvement often require aggressive oncological resection of large segments of the oral cavity including the mandible. These patients require vascularized donor osseous free tissue transfer to reconstruct significant defects. Traditionally, the donor bone is harvested on its vascular supply and shaped to the defect in a free hand fashion (FHS). However, virtual surgical planning (VSP) has emerged as a method to optimize reconstructive outcomes and decrease operative time. The goals of this study are to assess superiority of VSP to FHS by comparing bony union rates at 12 months, short and long-term complication rates, reconstruction accuracy, quality of life (QOL), functional outcomes, and economic analysis.

**Methods:**

This is a multicenter phase II/III study randomizing four hundred twenty head and neck patients undergoing mandibulectomy in a 1:1 ratio between VSP and FHS. Intention-to-treat analysis will be performed for patients enrolled but unable to undergo VSP-aided reconstruction. The primary endpoint is bony-union rates at 1 year post-operatively. Secondary outcomes include complication rates, QOL, functional outcomes, and economic burden.

**Discussion:**

This study will provide an assessment of two different surgical approaches to the reconstructive methods of mandible defects using fibular or scapular free flaps on bony-union rates, complications, QOL and economics.

**Trial registration:**

Clinicaltrials.gov identifier: NCT05429099. Date of registration: June 23, 2022. Current version: 1.0 on March 6, 2024.

**Supplementary Information:**

The online version contains supplementary material available at 10.1186/s12885-025-13505-5.

## Background

Vascularized bony free tissue transfer is required to reconstitute mandibular defects caused by extirpation of advanced benign or malignant neoplasms. The fibular free flap (FFF) was first introduced in 1989 by Hidalgo [1] and is the workhorse donor bone for reconstruction of mandibular defects due to its dependable vascular anatomy, ability to perform multiple osteotomies, and ample bony stock for dental implant support. More recently, the scapular free flap (SFF) has gained popularity for mandibular reconstruction due to its reliable pedicle length and diversity in composite flap options including bone, muscle, and multiple skin islands [2]. The harvested donor bone often requires multiple osteotomies in complex 3-dimensional (3D) orientations in order to reconstitute the premorbid mandibular anatomy. Traditionally, the donor bone was harvested in a free hand fashion (FHS), where once the vascular anatomy is isolated and ligated, the donor bone is iteratively cut into one or more segments to recreate the curved contour of resected mandible. Within a reasonable ischemia time, each segment must be accurately shaped to recreate the mandibular contour, while preserving its periosteal blood supply, and maintain good contact with its opposing donor bone or mandibular segment to facilitate bony union. Reported complications rates of non-union are 5–28%, and of plate exposure are 3–46% [3–13]. The accuracy of the reconstruction, and the ability to achieve union has significant impact on cosmetic and functional outcomes including bite force, return to a normal diet, and dental rehabilitation and ultimately quality of life [4, 14–19]. 

Recently, virtual surgical planning (VSP) has emerged as a method to improve reconstructive outcomes with evidence of decreased operative time and improved accuracy and union rates [20–25]. VSP leverages preoperative CT scans of the mandible and donor bone in a controlled setting with quantitative and qualitative feedback to virtually plan the reconstruction. Once a reconstruction is chosen, customized surgical cutting guides are then 3D printed, sterilized, and brought to the operating room and applied intraoperatively to the mandible and donor bone. There are a plethora of observational and retrospective studies supporting the benefits of VSP to improve operating room efficiency and patient outcomes [26–35]. A recent retrospective study comparing 43 FHS patients with 49 VSP patients demonstrated a significantly improved rate of non-union of 18.6% vs. 4.1% respectively [36]. A meta-analysis by Tang et al. (2019) [27] reported a standardized decrease of 1.55 h (95% CI: 1.22 to 1.87, *p* < 0.001) of ischemia time to revascularize the bone graft (8 trials included) and 1.01 h (95% CI: 0.80 to 1.23, *p* < 0.001) in total operative time with VSP. *However*,* there are no RCT studies comparing union rates*,* functional outcomes*,* or long-term outcomes of in-house VSP vs. FHS for mandible reconstruction with the fibula or scapula free flap*,* leading to a lack of evidence of superiority of one method to another.*

Given the lack of consistency amongst Canadian health authorities and US insurance plans in terms of the adoption of VSP, a randomized trial is critical to guide optimal reconstructive strategies. The goal of this randomized phase II/III study is to formally compare VSP to FHS, while also informing the design for optimal VSP strategies.

## Methods/design

This protocol, consent form, and data collection forms have been approved by the Research Ethics Board (REB) at the University of British Columbia (H20-03314), in compliance with the Helsinki Declaration.

### Objectives

The primary objective of the proposed trial is to determine if mandibular reconstruction using VSP results in better bony union rates assessed with postoperative CT scans at 12 months compared to standard FHS reconstruction techniques. Secondary objectives are to compare other short and long-term complication rates, reconstruction accuracy, quality of life, and functional outcomes of VSP and FHS. An economic analysis of VSP will also be performed.

### Primary outcome

- The primary outcome is non-union as assessed by two independent radiologists at the Vancouver General Hospital (VGH), blinded to the intervention, based on the 12-month postoperative CT scan. Each apposition (between native bone and the donor bone, or between flap segments) will be assessed as non-union, partial union, and complete union. Cases with a disagreement between reviewers will undergo consensus review, and any persisting disagreements will be reviewed by a third radiologist and classification will be based on the majority vote. The non-union rate was chosen as the primary outcome due to the clinical impact of non-union on the potential development of wound complications [10, 19]. As well, based on a retrospective review of non-union data at the VGH, there is a significant difference in non-union rate between patients who underwent FHS and VSP facilitated oromaxillofacial reconstruction.

### Secondary outcomes

- Complication rates: These include short-term and long-term complications such as flap failure, infection, and long-term plate extrusion. Adverse events will be identified during routine follow-up, by chart review and by a structured interview with participants.
- Operative time, ischaemic time and length of stay will be abstracted from the patients’ medical chart.
- Structural reconstruction accuracy: These will be measured comparing preoperative versus postoperative CT scans using cephalometric measurements including intracondylar distance and mandibular angles.
- Quality of Life measurements: The University of Washington Quality of Life Questionnaire (UWQOL), MD Anderson Dysphagia Inventory (MDADI), and The General Oral Health Assessment Index (GOHAI) will be collected to be consistent with other studies in this patient population [36, 37]. The UWQOL is a well-validated instrument for this patient cohort and has been demonstrated to have strong internal consistency (Cronbach’s alpha = 0.86) and reproducibility score of > 0.9035 [38–40]. The MDADI has high internal consistency (Cronbach alpha = 0.96) and test-retest stability ranging from 0.69 to 0.8837 [40].
- Functional Outcomes:
  - Occlusal force (bite force): will be determined using the Dental PreScale System (DPS–Fujifilm Global).
  - Donor site morbidity: will be measured by the Lower-Limb Tasks Questionnaire (LLTQ) or the Disabilities of the Arm, Shoulder, Hand (DASH) Questionnaire, both of which have been validated for patients receiving surgery affecting these areas [41, 42].
  - Occlusion and jaw freedom of movement will be measured and recorded.
  - Dental implantability will also be assessed and determined post-operatively by an independent oral surgeon, who will be blinded to the interventions on the trial [43].
- Economic Analysis: This will be performed based on the EQ-5D-5 L survey and a health utilization questionnaire (HUQ). Non-hospital visits will be captured through the HUQ that will be administered during each data collection point. Costs of all health care encounters will be done by usual means (e.g., physician costs from Provincial fee schedules, hospital costs from hospital finance departments, drug costs from the health authority and Provincial schedules). We will examine societal costs through the HUQ which has a section on lost productivity and other ‘out-of-pocket’ costs.

#### Inclusion criteria

- Primary diagnosis requiring mandibulectomy with fibular or scapular free flap reconstructive surgery.
- Individuals over the age of 18.
- Cognitive ability and language skills that allow participation in the trial.
- Ability to provide informed consent.

#### Exclusion criteria

- Contraindications to surgery such as severe comorbidities including metastatic disease.
- Individuals who do not have a recent CT scan (at most 30 days prior to their surgery), or individuals who are unable or unwilling to obtain a CT scan at most 6 days prior to their surgery, in order to allow time for guide design and sterilization in-house at VGH.
- Pregnant or lactating individuals.

### Interventions

Both patient groups will receive the standard presurgical work-up including CT imaging, quality of life questionnaires, functional evaluation of both donor sites, and a functional evaluation of bite force and jaw mobility.

**Fig. 1:**
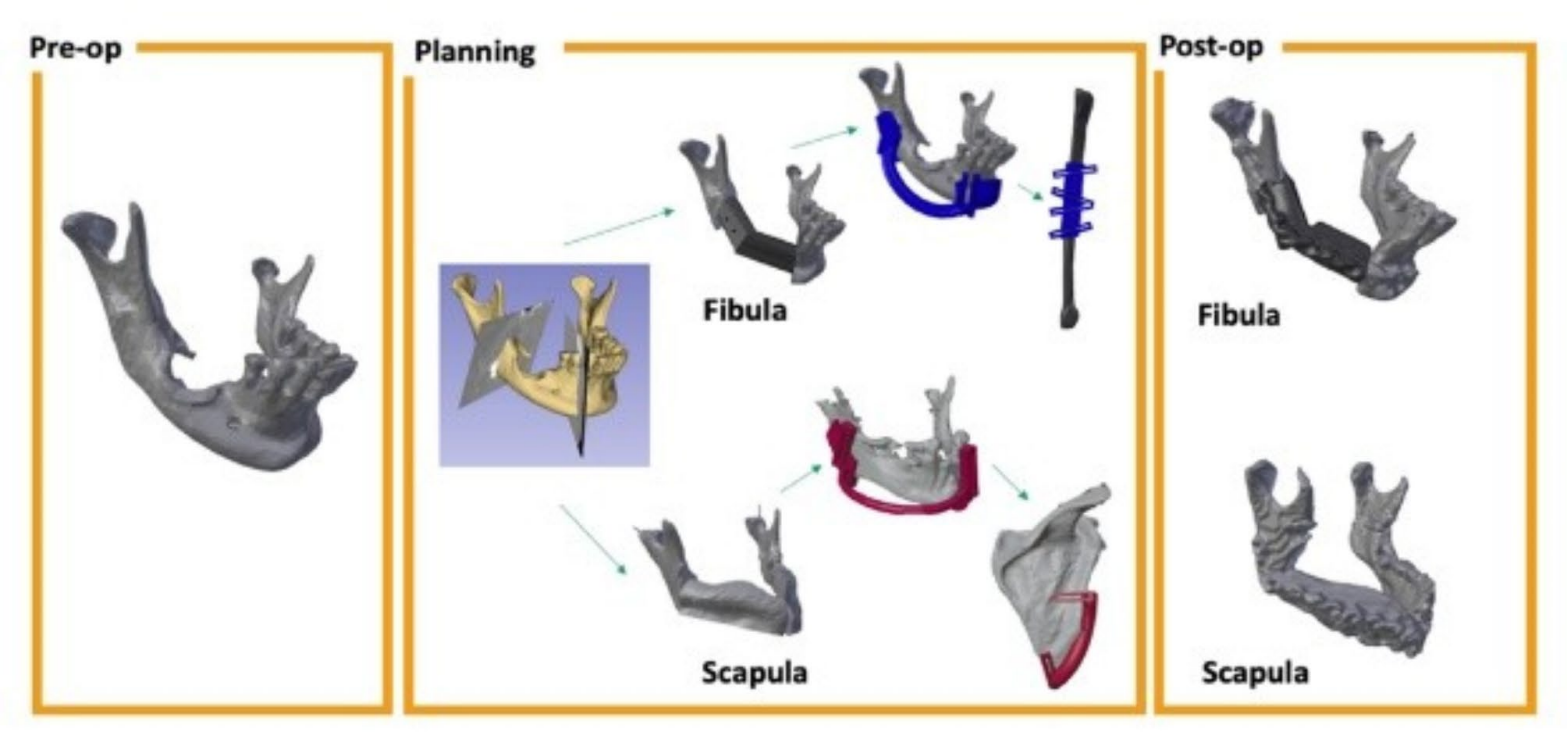
Overview of the VSP workflow. Pre-op: Segmentation of a diseased mandible. Planning: Cutting planes virtually defined on the diseased mandible. Reconstructions are planned using the fibula (top half) or scapula (bottom half). The planned reconstruction, the customized mandible cutting guide and the custom fibula/scapula cutting guides are shown. Post-op: segmentation of the post-operative CT of the reconstructed mandible

**Fig. 2:**
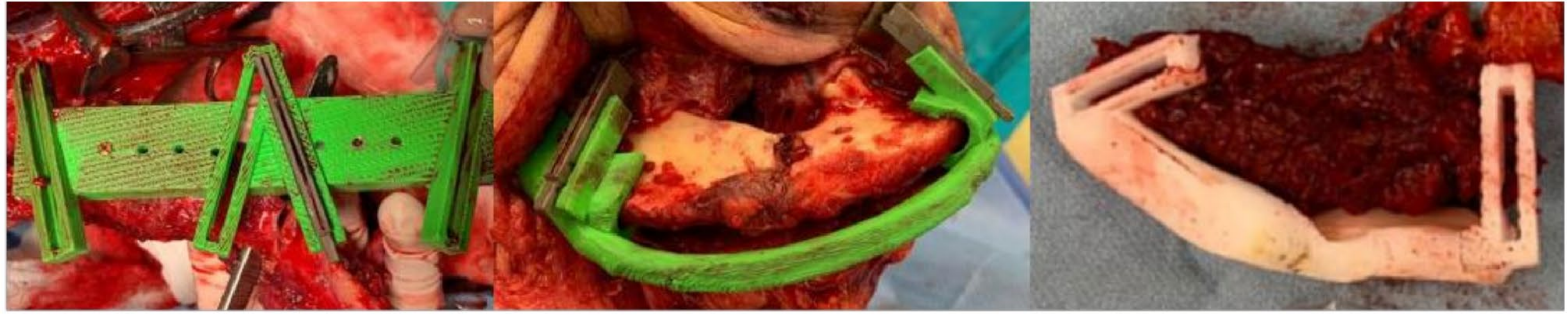
Intraoperative use of fibular cutting guide (left), mandibular cutting guide (centre) and scapular cutting guide (right)

Experimental arm - VSP: The trial research engineer (RE), located at VGH, will segment the CT data to create a 3D model for surgical planning. During the teleconference between site surgeon (SS) and RE, the RE will load the CT data and the segmented 3D model into the virtual planning environment. With the RE navigating the software, which was created in-house at VGH and used in a previous case series, the SS will determine the extent of the disease and define the resection planes [44–51]. After the cutting planes are created, the RE will use the software to create the reconstruction plan with either the patient-specific fibula or scapula, from which the donor bone will be chosen based on the reconstructive cephalometrics. Once the surgeon is satisfied with the plan, the teleconference will end and the RE will create and 3D print the surgical guides for the mandible, for the fibula or scapula, as well as the 3D computed reconstruction (Fig. 1). The surgical cutting guides will be sterilized at VGH’s Medical Device Reprocessing Department and couriered to the SS. The 3D reconstruction model, not requiring sterilization, will be sent directly to the SS. Prior to surgery, the SS will prebend a titanium fixation plate to the reconstruction model and send it for sterilization at the host’s hospital. The planning process, including shipping, will take at most 6 business days. The SS’s medical office assistant is responsible for receiving the shipments and transferring them to the SS. During surgery, the SS will employ the cutting guides and pre-bent titanium plate (Fig. 2). The mandibular cutting guides will be used to guide the resection, and the pre-bent plate is then applied to the remaining native mandible. Next, either the fibular or scapular cutting guide is applied to harvest the donor bone, which is then secured to the plate, followed by re-vascularization of the flap at the head and neck. If the planned resection cannot proceed (possibly due to extensive tumour growth since the time of surgical booking), the surgical team will note the reason for abandonment and conduct a standard FHS.

All SS will attend an initial ex-vivo training workshop hosted at VGH, plan a prospective VSP case, and observe the SS-VGH perform a VSP surgery. Each participating site must demonstrate the successful completion of VSP for 5 cases before randomizing patients in the trial. The SS all have prior experience with the VSP process through their medical training. The training workshop and 5-case requirement allows the SS to identify specific issues associated with VSP workflow at their sites. Based on the number of patients eligible for recruitment to the study, we estimate that the 5-case requirement will take at most 6 months to complete.

**Fig. 3:**
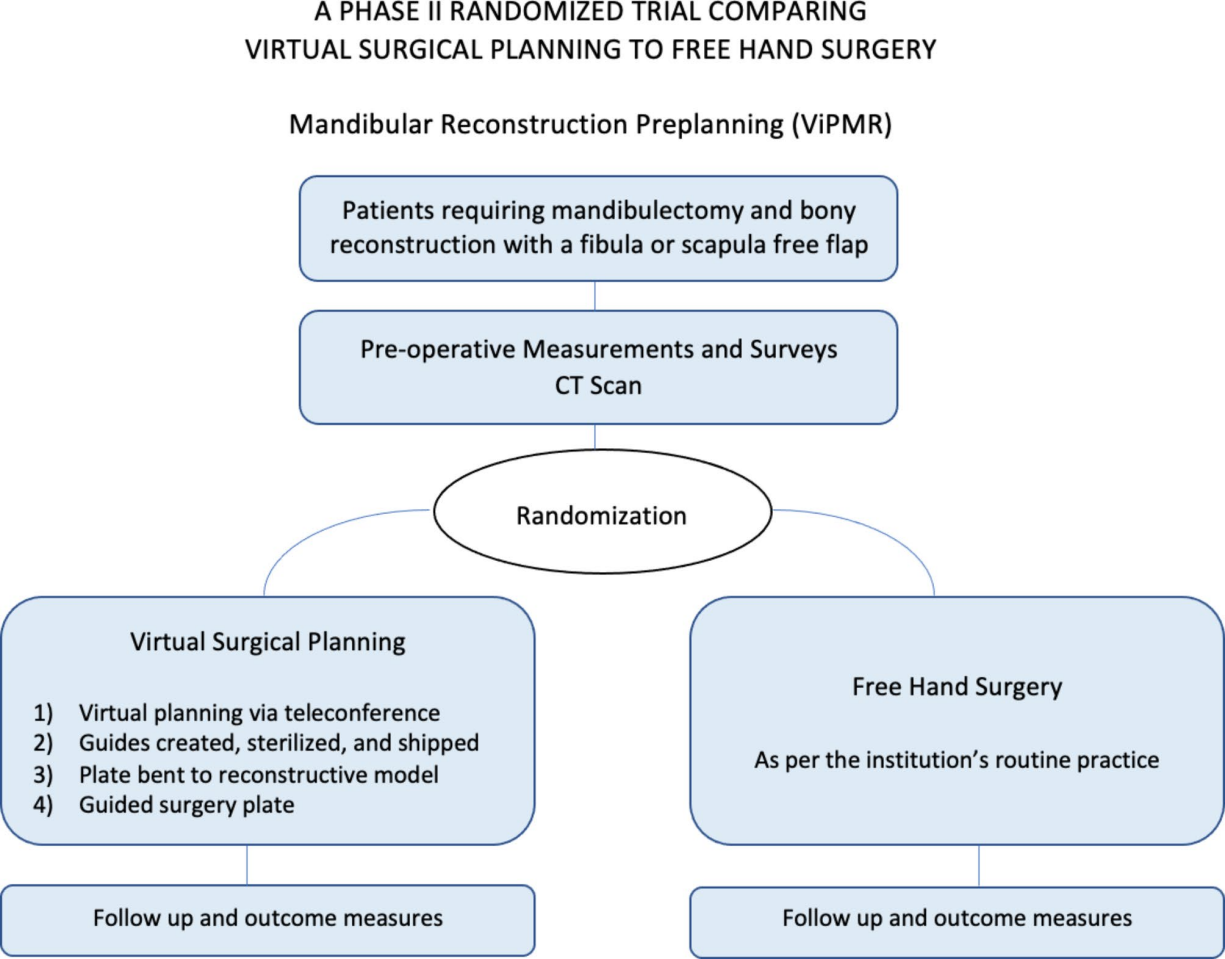
Study schema

Control arm - FHS. In FHS, the SS will proceed with surgery as per the institution’s routine practice. The donor bone will be selected based on clinical and radiological presentation, along with a patient preference-based clinical discussion (Fig. 3).

### Participating centers

The 7 centers include Vancouver General Hospital (Vancouver, BC), Royal Jubilee Hospital (Victoria, BC), London Health Sciences Center (London, ON), Sunnybrook Hospital (Toronto, ON), St. Joseph’s Hospital (Hamilton, ON), University Health Network (Toronto, ON), and Ottawa Hospital (Ottawa, ON).

### Allocation: sequence generation and implementation

The surgeons at each site will screen all patients for eligibility at the time of their preoperative appointment. Patients who express an interest in participating will meet with the local site research coordinator (LSRC) during the same visit to review the study. If the patient consents at the time of the clinic visit, all baseline data collection will be done at that time. If the patient consents after the visit the LSRC will arrange for a clinic visit to collect the baseline data. After baseline data collection is complete, the baseline CT scan will be uploaded to the REDCap database and reviewed by the research engineer. After confirmation of eligibility, the RE will access REDCap to randomize the patient. Randomization will be centralized, web-based and concealed using the randomization function within REDCap. Patients will be randomized in a 1:1 ratio. Treatment allocation will be stratified by hospital site and disease type: noncancer/cancer. Permuted blocks of varying sizes will be employed and will be unknown to study personnel. An independent statistician will create the randomization list and upload the list to the REDCap database.

This is a single-blind study as the SS will not be blinded to the intervention since they will be applying the guides during the reconstruction surgery procedure. After the randomization list is uploaded onto REDCap, the LSRC who will be performing the data collection, the radiologists who will be performing the bony union analysis, the oral surgeons/prosthodontists who will be performing dental rehabilitation assessments, and the statistician and statistical graduate students who will be performing the statistical analysis will not have access to the list as they will be blinded to the intervention.

### Treatment and follow-up

Patients will have their surgery scheduled according to local practice. Those patients randomized to the VSP arm will need a minimum of 6 business days from consent to surgery to allow for the production and shipping of the surgical guides. A comparable delay in the FHS arm will not be present, however most patients wait on average 3–4 weeks between consent and surgery.

Participants will be followed during their hospital stay (on average 7–10 days) and at standard clinic intervals (1-, 6-, and 12- months post-surgery) (Table 1).

**Table 1:** Schedule of events

Schedule of events	Enrollment			Follow-up				Close-out
	Baseline visit	Allocation	Before surgery	5 days	1 m	6 m	12 m	
Screen for eligibility	X							
Informed consent	X							
Allocation		X						
Screen for adverse events					X	X	X	
Head and neck; abdominal or leg CT scans			X (at least 6 days prior to surgery)	X			X	
Virtual surgical planning; guides 3D printed, sterilized, and shipped to participating sites			X					
Clinical Measurements (jaw mobility, bite force)	X				X	X	X	
Surveys (UW-QOL, EQ-5D-5 L, MDADI, LLTQ/DASH, GOHAI) and HUQ	X				X	X	X	
Data analysis								X
Publication								X

The patient population under study (60% squamous cell carcinoma, 10% ameloblastoma, 7.5% osteosarcoma, 15% osteoradionecrosis, and 7.5% other) have a serious illness and are largely compliant with scheduled follow-up. To maintain desired precision (power), we have incorporated a 5% loss to follow up in our sample size calculation. We anticipate 10% will die within the 1st year of surgery based on the AJCC staging manual and historical site data [52]. Deaths will be considered a competing risk to non-union in the analysis.

### Statistics and sample size calculation

The primary estimand of interest is the between-group difference in the proportion of trial patients who have a nonunion at 1 year. Point and interval estimates for the between-group difference at 1 year will be determined using the Fine-Grey competing risk regression model with treatment group as a covariate and a robust standard error to account for within-centre homogeneity of the outcome. Participants will be analyzed according to their treatment allocation regardless of treatment received (treatment policy perspective). In the primary analysis we assume that losses to follow-up (censoring) are unrelated to outcome.

Assuming 20% (FHS) vs. 10% (VSP) nonunion at 1 year, 15% mortality before 1 year, 5% loss to follow up before 1 year, beta = 0.20 and alpha = 0.05 (2-sided) yields a total of 420 randomized patients (210/arm). This sample size was determined based on 10,000 simulations incorporating the proposed analytic strategy. Across 7 participating centres and accounting for estimated loss to follow-up and mortality rates, we anticipate recruiting approximately 140 patients per year thus target recruitment should be met within 3 years.

### Statistical methods

Primary outcome: the primary estimand of interest is the between-group difference in the proportion of patients with a nonunion at 12 months in the full trial population grouped according to their treatment allocation (treatment policy estimand). Point and interval estimates for the between-group difference will be determined from a competing risk model that incorporates death as a competing risk.

Losses to follow-up will be censored at the last study visit. If the loss to follow up for the primary outcome is > 5% overall or in either treatment group, a sensitivity analysis will be conducted using a weighted generalized estimating equation approach. Secondary repeatedly measured, continuous outcomes will be analyzed using a linear mixed-effects model with a random effect for patient nested within study center. Structural accuracy, which has 4 measures: mandibular width and projection deviation, deviation in donor bone segment lengths and angles, will be compared with O’Brien’s global test to obtain an overall assessment of the intervention on the 4 accuracy measurements. O’Brien’s test is sensitive to effects that are consistent across the multiple measures increasing power, while simultaneously lowering type 1 error rates.

### Confidentiality

All hard copy records will be stored in a locked cabinet in the PI’s office, which is only accessible by the PI and authorized members of the research team. All data will be de-identified and labelled with a study ID that is not derived by personal identifiers, such as SIN, PHN, DOB or initials. A master list that will link the participant’s name and PHN to their study ID will be recorded on a hard copy that will be stored in the PI’s office in the Otolaryngology Clinic at VGH. This data will be kept separate from the data collection forms.

### Data and safety monitoring

The Data and Safety Monitoring Committee (DSMC) will consist of a head and neck surgeon with experience in clinical trials, a biostatistician and an expert in virtual surgery. The DSMC is independent from the investigators and team members involved in the trial. The DSMC will meet bimonthly to review any adverse outcomes and complication rates, and will recommend any modifications to the TSC. The DMSC must be sufficiently satisfied with study progress and safety to approve the continuation of the study.

An interim analysis will be performed by an independent statistician after 50% of the sample size have enrolled and completed their 1-year follow up and CT scan. The statistician will report to the DSMC, which will then decide whether to continue the study based on the Peto stopping guideline (two-sided *p* < 0.001). Data collected from the semi-structured interview will be reviewed during this analysis to determine whether sex, gender or other factors affect patient participation in the trial. The TSC will use this data to change recruitment strategy as appropriate.

### Adverse events

The Common Terminology Criteria for Adverse Events (CTCAE) version 4.0 grading scale will be used to assess adverse events. For any grade 4 or 5 adverse event, the Principal Investigator and local REB will be notified, and a Serious Adverse Event form will be completed and stored on REDCap.

### Data storage & auditing

De-identified data will be stored on the secure online clinical database, REDCap. Access to REDCap will be granted to investigators, LSRCs, and RE. Independent from the investigators and researchers involved in the trial, the Vancouver Coastal Health Research Institute Clinical Research Unit will perform ongoing audits.

### Protocol amendments

Protocol amendments such as modifications to the eligibility criteria, outcomes, analyses will be approved by the principal investigator and will be communicated to all investigators, REBs, and trial registries.

### Dissemination

Trial results will be embargoed until information release is authorized by the principal investigator and will be communicated to the public, participants and healthcare professionals via publication and presentation within a year of study closeout. Authorship of the protocol and trial report will be decided by the principal investigator. We do not intend to use professional writers for any stage of manuscript preparation.

## Discussion

Despite advances in surgical techniques, the morbidity associated with advanced oral cavity defects requiring reconstruction remains high, and its subsequent detrimental effects on quality of life are well documented [53]. VSP has great potential to reduce complication rates, limit morbidity, and improve functional outcomes and quality of life; however, there is no high-quality evidence to support its efficacy. As a result, VSP adoption is variable, with scepticism concerning clinical outcomes and cost-effectiveness. In Canada, three centres have already adopted this technique including Misericordia Community Hospital (Edmonton), Foothills Medical Centre (Calgary) and Montreal General Hospital. However, two of the three centres utilize commercial VSP products with significantly increased costs to the health care system. The third centre has a well-funded program that provides an in-house solution that could only be translated to other centres with significant capital cost investments in software and hardware.

The in-house VSP technology proposed in this study is open-source and can be easily adopted by other centres. The remaining centres across Canada, including those enrolled in this trial, account for over 50% of total mandibular reconstructions in the country and have not adopted VSP due to a lack of definitive evidence of its benefits and its significantly increased cost. A recent base-case analysis by Fatima et al. (2019) [54] found VSP was costlier than usual care by $7099.00 (2016 US $) per person. Furthermore, their analysis revealed higher flap loss (83/1000 vs. 77/1000) and higher rates of mandibular infection (308/1000 vs. 243/100) with VSP vs. FHS, although considerable uncertainty was associated with these estimates [54]. This contrasts various retrospective series demonstrating increased operating room efficiency and structural outcomes [27]. 

ViPMR is a multi-center phase II/III trial that aims to randomize 420 patients to two reconstructive interventions, VSP vs. FHS, in order to assess the superiority of VSP over FHS particularly as it relates to union rates at 1 year post treatment. Additional economic, structural and functional outcomes will also be collected to determine if VSP can contribute to improve outcomes for advanced oral cavity malignancies in a financially responsible manner. Results of this study will help guide Canadian health authorities and US insurance plans in terms of the adoption of VSP nation-wide.

## Electronic supplementary material

Below is the link to the electronic supplementary material.

Supplementary Material 1

## Data Availability

No datasets were generated or analysed during the current study.
