# Supplementary material for: A multi-centre, participant-blinded, randomized, 3-year study to compare the efficacy of Virtual Surgical Planning (VSP) to Freehand Surgery (FHS) on bony union and quality of life outcomes for mandibular reconstruction with fibular and scapular free flaps: study protocol for a randomized phase II/III trial
Source: BMC Cancer. 2025 Feb 27;25:358. doi: 10.1186/s12885-025-13505-5 (PMC11866805; doi:10.1186/s12885-025-13505-5)
Supplement: Supplementary file 1 — Supplementary Material 1 [file 12885_2025_13505_MOESM1_ESM.docx]

# World Health Organization Trial Registration Dataset

| **Item** | **Description** |
| --- | --- |
| Primary registry and trial identifying number | ClinicalTrials.gov  NCT05429099 |
| Date of registration in primary registry | June 23, 2022 |
| Secondary identifying numbers | NA |
| Source(s) of monetary or material support | Terry Fox Research Institute  Vancouver General Hospital-University of British Columbia Hospital Foundation  Canadian Institutes of Health Research  Michael Smith Foundation for Health Research |
| Primary sponsor | Vancouver Coastal Health Research Institute |
| Secondary sponsor(s) | NA |
| Contact for public queries | Dr. Eitan Prisman |
| Contact for scientific queries | Dr. Eitan Prisman |
| Public title | A randomized trial to compare virtual surgical planning and freehand surgery for lower jaw reconstruction |
| Scientific title | A multi-centre, participant-blinded, randomized, 3-year study to compare the efficacy of Virtual Surgical Planning (VSP) to Freehand Surgery (FHS) on bony union and quality of life outcomes for mandibular reconstruction with fibular and scapular free flaps: study protocol for a randomized phase II/III trial |
| Countries of recruitment | Canada |
| Health condition(s) or problem(s) studied | Mandibular reconstruction with the fibular or scapular free flap; oral cancer or osteroradionecrosis |
| Intervention(s) | Active arm: virtual surgical planning; control arm: free hand surgery |
| Key inclusion and exclusion criteria | Inclusion Criteria: 1) primary diagnosis requiring mandibulectomy and fibular or scapular free flap reconstructive surgery, 2) are over the age of 18, 3) cognitive ability and language skills that allow participation in the trial, 4) provide informed consent  Exclusion Criteria: 1) severe comorbidities including metastatic disease; 2) do not have a recent (within 30 days) CT scan and are unable/unwilling to receive a head CT scan at the latest 6 days prior to surgery |
| Study type | Interventional (clinical trial)  Allocation: randomized  Interventional model: parallel assignment  Masking: participant-blinded  Primary purpose: treatment |
| Date of first enrolment | October 1, 2022 |
| Target sample size | 420 |
| Recruitment status | Recruiting |
| Primary outcome(s) | Bony union rate at 12 months post-operative |
| Key secondary outcomes | Short and long-term complication rates, reconstruction accuracy, quality of life, and functional outcomes of VSP and FHS, economic analysis |
| Ethics Review Status | Approved |
| Date of approval | August 24^th^, 2021 |
| Name and contact details of Ethics committee | UBC Clinical Research Ethics Board (Sarah Flann, sarah.flann@ubc.ca) |
| Completion Date | Recruiting |
| Summary Results | Recruiting |
| IDP Sharing | Plan to share IDP: Yes |
| Agreement | Plan description: Qualified researchers conducting independent scientific research may submit requests to access trial IDP after publication of results.  Anonymized IDP will be made available for 12 months after review and approval of the study’s protocol and completion of a data sharing agreement. |
